# Have proton exchange membrane fuel cells been designed for recycling?

**DOI:** 10.1177/0734242X251360546

**Published:** 2025-08-18

**Authors:** Samuel D Widijatmoko, Yichang Yan, Qiqiu Huang, Shangfeng Du, Yongliang Li, Gary A Leeke

**Affiliations:** School of Chemical Engineering, University of Birmingham, Birmingham, UK

**Keywords:** PFSA, GDL, recovery, direct recycling, fuel cell, Nafion, platinum

## Abstract

Considering the widespread use of proton exchange membrane (PEM) fuel cells, end-of-life management is critical. The main component of PEM fuel cells is the membrane electrode assembly (MEA), comprising a Nafion membrane sandwiched between two electrodes. The electrodes are made of platinum supported on carbon (Pt/C) and held together by the Nafion binder. Researchers have mainly focused on the recovery of platinum from the MEA. The low concentration of platinum in the electrodes makes thermal decomposition of the fluorinated polymeric binder, followed by a hydrometallurgy step being the most practical way to liberate and concentrate the platinum catalyst. In this manuscript, we show the use of aqueous ethanol and isopropyl alcohol to swell the PEM and recover it intact. The solvent mixture containing the electrodes is then subjected to microwave heating to dissolve the Nafion binder and liberate the Pt/C which can be further enhanced with induced mixing. This approach offers the potential for MEA short loop recycling. The proposed approach concentrates 87% of the platinum catalyst while being able to produce clean Nafion membrane. Finally, we show that the recycled gas diffusion layer can be re-used to create a new MEA with a slight decrease in performance.

## Graphical abstract

**Figure:**
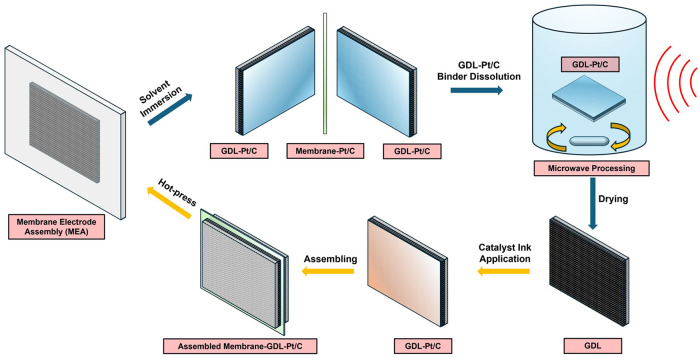

## Introduction

Proton exchange membrane (PEM) fuel cells are a promising low carbon technology (Cullen et al., 2021). It is anticipated that there would be 400 million fuel cell-powered vehicles by 2050 (Halder et al., 2024). The main part of a PEM fuel cell is a membrane electrode assembly (MEA) that comprises a perfluorinated sulphonic acid-based (PFSA) polymer electrolyte membrane (e.g. Nafion) sandwiched between an anode and a cathode (Uekert et al., 2023). The electrodes in a PEM fuel cell are composed of a catalyst layer (CL, i.e. platinum supported on carbon (Pt/C)) and a gas diffusion layer (GDL). The fabrication of a CL is typically done by spray-coating a Pt/C and Nafion mixture dispersed within an aqueous solvent, often referred to as the catalyst ink, onto a GDL to make a gas diffusion electrode or membrane surface to make the catalyst coated membrane (CCM; Sgarbi et al., 2023; Turtayeva et al., 2022). The GDL is composed of a microporous layer, usually carbon particles bound with polytetrafluoroethylene (PTFE), and a macroporous substrate made by carbon fibre (Park et al., 2006). The microporous layer of the GDL in the fuel cell assembly interfaces with the CL, while the macroporous layer interfaces with the gas flow field (Park et al., 2012). An elevated temperature (ca., 80°C–140°C) hot press is usually used to bond the CCM and the GDL (Najafi Roudbari et al., 2017). To make a complete PEM fuel cell, the MEA is then sealed in between a pair of current collectors and bipolar plates (Pollet et al., 2016). The MEA represents 60–73% of the manufacturing cost of PEM fuel cells with the high cost being attributed to the platinum catalyst (59%) as well as the PEM (11%; Kleen et al., 2023; Yang, 2015). With the growing popularity of PEM fuel cells, it is important to consider the fate of the fuel cell at the end of life.

PEM fuel cells recycling has mainly been focused on the recovery and recycling of platinum catalysts from the MEA (Duclos et al., 2017; Trinh et al., 2024). However, components such as current collectors and bipolar plates are easily recoverable by simply unscrewing the PEM fuel cell stack. Hydrometallurgical and pyrometallurgical processes being the main routes which only target the platinum (Barakat and Mahmoud, 2004; Dong et al., 2015; Duclos et al., 2016). However, to minimize the environmental impact of the PEM fuel cell technology, Nafion and GDL recovery and recycling should also be addressed (Bauer et al., 2024; Valente et al., 2019). The research community in PEM fuel cells has set a target to reduce the platinum catalyst loading from 0.365 to 0.125 mg cm^−2^ for recycling to be economically viable (Wang et al., 2022). It is therefore expected that the yield of platinum from recycling end-of-life PEM fuel cells will decrease over time, with the prospect of deriving value from components other than catalyst becoming important. This has led to investigations into alternative pre-treatment processes that also allow the recovery of the GDL and PEM. One strategy is to take advantage of the swelling of the PEM with aliphatic alcohols to induce separation between the PEM and the electrode (Fu et al., 2008). Using aqueous ethanol (EtOH) or isopropyl alcohol (IPA) results in good separation of the Nafion membrane with further cleaning needed, which typically done with induced mixing or sonication (Oki et al., 2009; Shore, 2012). Novel flow-based cleaning of the Nafion membrane with aqueous alcohol mixture has also been proposed (Carmo et al., 2019). More recently, Robert et al. (2025) compared the use of EtOH and IPA to recover the different components of MEA at slightly elevated temperature (ca., 40°C–75°C) with the optimum temperature for delamination and dissolution in 50% IPA found to be 40°C and 75°C, respectively. This approach ensures the Nafion used as membrane and catalyst binder remain separate throughout the recycling process. Nevertheless, the recovered GDL was not evaluated.

Substantial progress has been achieved in optimizing the recovery value of MEA components, extending beyond platinum recovery. Using aqueous alcohol to swell and separate the MEA into different components is a promising approach. However, the focus only incorporates the recycling of the PEM with limited research that has investigated the induced mass loss as well as GDL quality after treatment. The assessment of GDL after treatment such as swelling and dissolution is important since the ionomer used for PEM is selected of different types to that of the catalyst binder (Robert et al., 2025). As the swelling behaviour of polymer is type-dependent (Jalani and Datta, 2005), differences in swelling characteristics of the membrane and ionomer binder could lead to CL presence on both the Nafion membrane and GDL surfaces following solvent-induced swelling.

Although recent studies suggest that the dissolution of Nafion can be done to liberate the platinum catalyst, no means of assessment regarding the Nafion mass loss, the remaining Nafion binder on GDL, and the potential recovery of platinum was made. In addition, despite the thorough analysis presented by the previous studies implying the opportunity for direct recycling, the actual MEA made from the recovered GDL has never been assessed for re-use. Therefore, this study aims to recover the Nafion membrane and the GDL at the highest possible value by using green solvents (water, EtOH and IPA). Initially, this study explores how EtOH and IPA concentrations impact MEA separation and Nafion membrane cleaning via ultrasonication. The Nafion mass loss due to sonication is then briefly discussed. The electrodes are then subjected to further treatment to liberate the Pt/C catalyst by Nafion binder dissolution using microwave heating. The amount of Nafion binder on the GDL microporous surface is qualitatively compared using Fourier transform infrared spectroscopy–attenuated total reflectance (FTIR-ATR) with a germanium ATR crystal. Finally, we demonstrate the reusability of recycled GDL to make new MEA for the first time.

## Experimental

**Figure 1.**
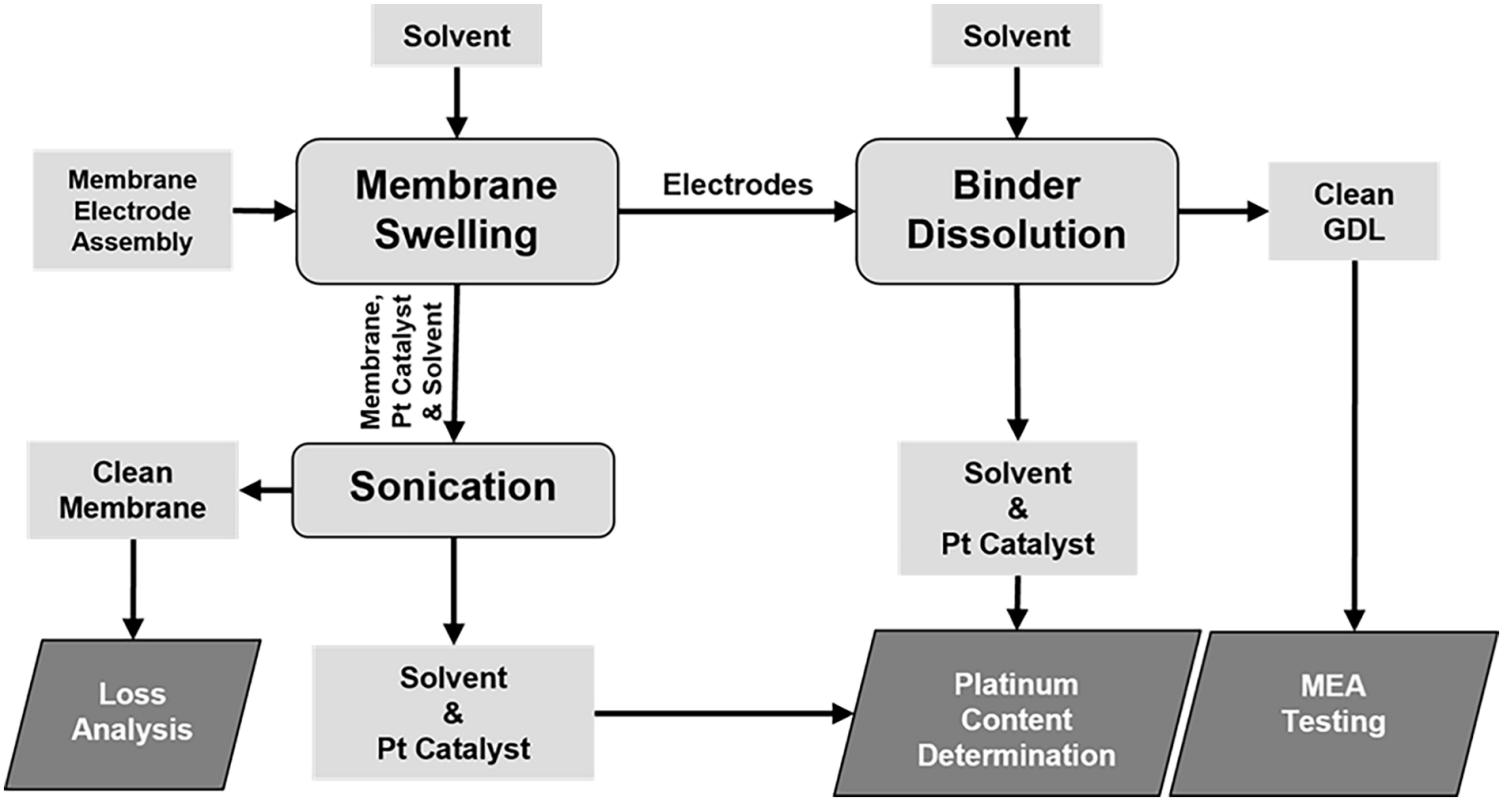
Flow diagram of the experimental process.

Clean Nafion membrane and GDL were obtained by a three-step procedure: (1) MEA separation by Nafion membrane swelling; (2) Nafion membrane cleaning by sonication and (3) Nafion binder dissolution from the GDL microporous layer. The flow diagram of the whole experimental process is summarized in Figure 1.

### Membrane electrode assembly

The Birmingham Centre for Fuel Cell and Hydrogen Research provided the used MEA samples used in the study. All samples used in the study were of industrial grade and were made in 2019. The Nafion membrane and electrodes dimensions are 6 × 6 and 4 × 4 cm, respectively. This study used samples with a platinum loading of 0.2 mg cm^−2^.

### Membrane and electrode separation via solvent swelling

Double-distilled water was used throughout this study. EtOH (analytical reagent grade; Fisher Scientific, UK) and IPA (HPLC grade; Sigma-Aldrich, Merck UK) was mixed with water by weight to create the working solvent. In a 100-mL beaker, a cut 0.5 × 0.5 cm MEA sample was immersed in 20 mL of solvent for 5 minutes. The detached electrodes were manually recovered with tweezers and subsequently dried overnight at 70°C in an oven (Binder™, Binder GmbH Germany). The resulting dried electrodes are subjected to FTIR-ATR (Nicolet Summit X, Thermo Scientific USA) with germanium ATR crystal. All spectra were recorded between the wavenumber of 600 and 4000 cm^−1^ with 16 scans and a resolution of 4 cm^−1^. The beaker with the detached membrane was then transferred to a 15-L ultrasonic bath (30 kHz; Langford Sonomatic, UK) to remove the Pt/C adhering to the surface. Membrane cleaning was assessed based on visual inspection while still in the solvent to prevent shrinkage, and the sonication time was varied to achieve the desired cleaning. Throughout the experiments, the temperature of the ultrasonic bath was kept below 40°C monitored with infrared thermometer gun (RS Pro, RS-8876; RS Components UK). The membrane was then transferred on to a PTFE sheet, patted dry with 125 mm filter paper (Fisherbrand, Fisher Scientific UK) and left to air-dry overnight prior to drying in the oven at 70°C. Before placing it on the PTFE sheet, the cleaned membrane was soaked in water for 5 minutes to decrease the likelihood of breakage through shrinkage and to enhance the robustness of the sample. The separation of the membrane and electrodes was assessed across a range of alcohol concentrations.

The sonication of the membrane could cause some Nafion to be dissolved into the alcohol water mixture. To evaluate this potential mass loss, a comparison was done by using a new 2 × 2 cm Nafion 211 membrane that has been protonated using concentrated HCl (ACS reagent; Sigma-Aldrich), rinsed with water and dried overnight in an oven at 70°C. Each run used approximately 0.02 g of membrane samples that were weighed to four decimal places using an analytical balance (Fisherbrand). The weighed membrane was processed using the previously described methods, then dried in a 70°C oven, and the mass change was recorded.

### GDL cleaning via binder dissolution

A GDL cleaning study was performed using solvents with varying alcohol concentrations, temperatures and holding times. At atmospheric pressure, the test was performed using a hotplate (Asynt – ADS-HP-NT and DrySyn^®^ MULTI-M) and 24 mm boiling tubes. At elevated pressure, the test was performed using 100-mL SK-15 rotor with a flexible microwave synthesis system (Flexiwave; Milestones, Italy). For each flask, both setups used 5 × 12 mm PTFE coated magnetic stirring bar and 15 mL of solvent. The hotplate system studies were conducted under reflux conditions with an impeller speed of 100 rpm; holding times were determined by visual inspection and a stopwatch. The timer was started once the emergence of gas bubbles was observed. However, for the microwave system, the mixing was set at 10%, with a temperature ramp rate of 10°C min^−1^ and the holding time set accordingly. Once the experiment finished, the system was allowed to cool to room temperature before retrieving the electrodes using a tweezer and dried in an oven at 70°C. To ensure comparability between each run, a reference was made. Electrodes used in the Nafion binder dissolution study came from samples that have been separated using 40% IPA.

FTIR-ATR spectroscopy was used to assess Nafion binder removal by comparing spectra from the GDL’s microporous layer before and after treatment, where the absorbance is directly proportional to concentration. The measurement was performed under ambient conditions, and the spectral data processing was done using the Thermo OMNIC Paradigm Software. A peak in the wavenumber region of 915 and 988 cm⁻¹ was used as a performance marker, as it has been shown to exhibit minimal sensitivity to changes in hydration (Kunimatsu et al., 2011). The processed absorbance (see Supplemental Figure S1A) in this region was used to compare the FTIR relative abundance of the Nafion between samples.

GDL morphology analysis was performed using a scanning electron microscopy with back scattered detector (SEM-BSD – Carl Zeiss EVO 10, Carl Zeiss Germany). The SEM-BSD was also equipped with energy dispersive X-ray (EDX; Oxford Instruments, UK) to identify the different elements present. The sample was mounted onto aluminium studs using a carbon tape with the surface made conductive using a carbon coater (Quorum Q 150T ES, Quorum Technologies UK) with the thickness programmed at 10 nm.

To better explain the cleaning efficacy, droplet contact angle measurement (Krüss, GmbH Germany) on the GDL was done with varying EtOH and IPA concentrations for a 4-µL droplet. A systematic screening was performed to find the alcohol percentage that allows instantaneous absorbance into the GDL. The initial screening utilised 10%, 20%, 40% and 80% alcoholic solutions. Subsequent fine-tuning steps using10% and 5% solutions were performed to determine the alcohol concentration that allows instantaneous absorbance into the GDL.

### Processing of larger sample and platinum content determination

The processing of larger samples used 40% IPA solution for the membrane swelling and Nafion binder dissolution. A beaker size of 600 mL was used for the membrane swelling and cleaning. The recovered electrodes were cut into a width of 2.7 cm to fit into the 100 mL SK-15 rotor. To ensure electrodes were fully submerged, 50 mL of the IPA solvent was used per rotor. The solution recovered from the membrane cleaning and binder dissolution was subjected to a vacuum rotary evaporator set at 55°C with 100 mL round bottom flask. Once the solvent had been evaporated, the sample was allowed to cool to room temperature. To digest the platinum, 25 mL of three parts 32% HCl and one part of 68% HNO_3_ mixture were added to the same round bottom flask. The solution was then diluted with 25 mL of water, followed by 3 hours reflux to dissolve the remaining platinum particles that may not have been previously immersed. The solution then let to cool down to room temperature, filtered, diluted and analysed using an inductively coupled plasma–optical emission spectroscopy (ICP-OES; Agilent 5110 VDV ICP-OES, USA). The platinum calibration curve was prepared by diluting platinum standard reference solution (Sigma-Aldrich).

### Direct recycling of the GDL

The recycled GDL was tested and compared to a new GDL (Sigracet 39BC, SGL Carbon GmbH Germany). The comparison was done by employing a catalyst loading of 0.2 mg Pt cm^−2^. The Pt/C catalyst (45.9% Pt; TEC10E50E) obtained from Tanaka Kikinzoku Kogyo K. K. was employed for both anodes and cathodes for both MEAs. About 2.5 mg of Pt/C catalyst was initially mixed with 40 μL of water followed by the addition of 160 μL of IPA. Subsequently, 14.4 μL of a 10% Nafion D1010 dispersion (Ion Power Inc., USA) was added. The resultant catalyst ink was sonicated for 10 minutes and then applied onto a 2.24 × 2.24 cm section of 39BC GDL paper under infrared illumination. Before the single fuel cell tests, a new Nafion 212 membrane was positioned between the prepared Pt/C electrodes and hot-pressed at 4.45 MPa for 2 minutes at 135°C.

Polarization curves and electrochemical impedance spectroscopy (EIS) were conducted using a Scribner 850e Fuel Cell Test Station, employing hydrogen at the anode and air at the cathode. All samples were hydrated for 60,000 seconds at 80°C with 100% relative humidity (RH) and 0.5 bar back-pressure before subsequent testing. The polarization curves were measured from 0.40 V to open-circuit voltage, with a scan step of 0.025 V at 80°C under 100% RH and 2 bar back-pressure. The potential at each point was maintained for 180 seconds. EIS measurements were performed over a frequency spectrum ranging from 10,000 to 0.1 Hz, using an amplitude corresponding to 10% of the direct current at a potential of 0.6 V.

## Results and discussion

### Membrane recovery and cleaning via solvent swelling and sonication

The membrane type was initially analysed using FTIR-ATR, and the spectrum can be found in the Supplemental Figure S2. The FTIR-ATR spectrum of the PEM, taken from the membrane, reveals peaks at 1212, 1153 and 1057 cm^−1^ which are the symmetric C–F stretching vibration, asymmetric C–F stretching vibration and symmetric S–O stretching vibration (Kunimatsu et al., 2011). The peaks at 982 and 967 cm^−1^ are the side chain asymmetric and symmetric ether, C–O–C, stretching (Hallinan et al., 2010). The peak assignment reveals a PFSA-based membrane, specifically Nafion (Agarwal et al., 2024). With the PEM type confirmed, we assessed the catalyst ink binder by exposing a chopped MEA to a 40% IPA solution to induce Nafion membrane swelling. FTIR-ATR analysis was performed on the recovered and subsequently dried electrodes. The spectrum obtained from the GDL’s microporous layer reveals peaks at 1209, 1151 and 1057 cm^−1^ that can be assigned to the symmetric C–F stretching vibration, asymmetric C–F stretching vibration and symmetric S–O stretching vibration, respectively (Kurniawan et al., 2013). The peaks at 981 and 968 cm^−1^ are the side chain asymmetric and symmetric ether, C–O–C, stretching (Choi et al., 2012). The GDL’s microporous layer is still coated therefore with the CL that is also of a Nafion-type PFSA. The slight discrepancy in the peak position can be attributed to the different level of Nafion hydration. Kunimatsu et al. (2011) reported that the symmetric C–F stretching vibration showed a significant shift from 1211 to 1221 cm^−1^ upon hydration. Based on the symmetric C–F stretching vibration, it can be understood that the Nafion on the GDL (1209 cm^−1^) is slightly more hydrated than the Nafion from the PEM (1212 cm^−1^) which may be caused by the difference in sample pre-treatment prior to analysis. Apart from the PEM and catalyst binder, analysis of the GDL’s macroporous layer revealed the use of PTFE as binder, evidenced by peaks at 1205 and 1150 cm⁻¹ corresponding to C–F stretching vibrations (Turk et al., 2024). In summary, the MEA sample used incorporates Nafion as both PEM and catalyst binder, with PTFE as the binder for the GDL.

**Figure 2.**
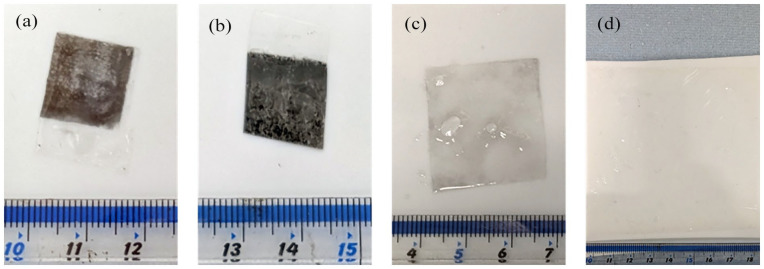
Photograph of samples following: (a) 15 minutes sonication in 40% EtOH, (b) 15 minutes sonication in 20% IPA, (c) 5 minutes sonication in 40% IPA and (d) 5 minutes sonication in 40% IPA and 5 minutes water immersion (full size). EtOH: ethanol; IPA: isopropyl alcohol.

In addition to verifying that Nafion is used as both a PEM and catalyst binder, we observed that during component separation the Nafion membrane swelled in the 40% IPA leading to at least double in area with some Pt/C catalyst found on the membrane. The study by Robert et al. (2025), showed that the particle detachment is mainly influenced by the solvent swelling capacity. It was thought that the catalyst can be concentrated on either side by inducing less membrane swelling or using different alcohol. A visual inspection, correlated with separation time, was used to analyse the swelling behaviour of EtOH and IPA at concentrations of 0%, 10%, 20% and 40%. Only IPA and EtOH above 20% allowed the separation of the Nafion membrane from the electrodes. Manual intervention by peeling off the GDL is required for 20% EtOH, whereas 40% EtOH, 20% IPA and 40% IPA did not require any intervention. The time taken for the separation to occur with 40% EtOH, 20% IPA and 40% IPA were 45, 102 and 24 seconds, respectively. The electrodes were then removed from the beaker and the Nafion membrane were soaked in solution for a total time of 5 minutes prior to sonication. During sonication, the membrane was visually inspected every 5 minutes. It was found that the sonication time and alcohol types affect the cleaning efficacy. Minimum cleaning was observed after 15 minutes sonication for 40% EtOH and 20% IPA solutions (Figure 2(a) and (b)). However, the 40% IPA solution facilitated cleaning within a 5-minute sonication timeframe (Figure 2(c)) and increasing the IPA concentration did not lead to better cleaning (Supplemental Figure S3). However, samples that have been treated with 40% IPA are less yielding and easier to break compared to the 40% EtOH and 20% IPA solutions. We observed that immersion in double-distilled water for at least 5 minutes facilitates easier handling and reduces the risk of the membrane being torn. Thus, the 40% IPA was found to be the optimum concentration.

The mass loss for Nafion membrane cleaning was undertaken with Nafion 211 and 40% IPA solution as well as to check whether the experimental protocol can be used for the recovery of larger membrane pieces without breaking. It was found that the Nafion membrane barely loses any mass (0.1% ± 0.4%) from swelling alone. For 5 and 10 minutes sonication, the respective mass losses were 2.0% ± 1.2% and 7.5% ± 0.4%. A 15-minute sonication weakened the membrane to the extent that it was challenging to recover without breaking and prevented precise quantification of mass loss. The outcomes of the mass loss analysis revealed an exponential rise in mass loss as sonication time extends, potentially causing membrane breakage and dispersion if not carefully monitored. Finally, successful recovery of the Nafion membrane with 40% IPA solution and 5 minutes sonication from a full-sized MEA was achieved (Figure 2(d)).

### Liberation of platinum catalyst via binder dissolution

Having shown that the Nafion membrane can be recovered as-is with minimum mass loss, the electrodes were further processed. Initially, we explored the possibility of using the same sonication technique to liberate the Pt/C adhering to the GDL. However, through visual inspection, PTFE-bound carbon fibre from the GDL’s macroporous layer was also liberated. Therefore, the GDL cleaning by Nafion dissolution was then proposed.

**Figure 3.**
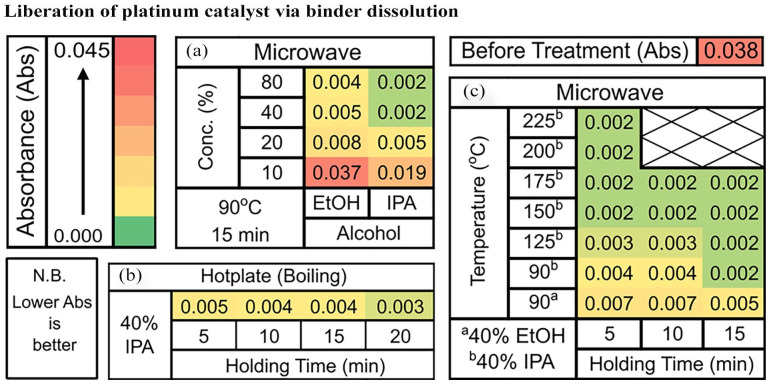
Heat map of FTIR-ATR absorbance of the GDL’s microporous layer at 980 cm^−1^ for the Nafion dissolution using a hotplate and a microwave reactor. (a) Nafion dissolution using a microwave reactor under varying EtOH and IPA concentration; (b) Nafion dissolution in boiling 40% IPA by using a hotplate; (c) Nafion dissolution using a microwave reactor at eleveated pressure and temperature. GDL: gas diffusion layer; FTIR-ATR: Fourier transform infrared spectroscopy–attenuated total reflectance.

The relative abundance of Nafion on the GDL’s microporous layer that has been subjected to different treatments were assessed by comparing the FTIR absorbance at 980 cm^−1^. The FTIR absorbance exhibits a direct, proportional relationship with Nafion concentration. The efficacy of a boiling 40% IPA solution (c.a., 83.3°C) in dissolving the Nafion binder across a range of holding time was investigated. With 5-minute holding time, the results from the FTIR-ATR analysis suggest a significant reduction in absorbance from 0.038 to 0.005 (Figure 3(b)). More Nafion can be removed from the GDL by increasing the holding time. Extending the holding time to 20 minutes leads to measured FTIR-ATR absorbance of 0.003. Unlike sonication, no PTFE-bound carbon fibre was seen to be liberated. Hence, the removal of Nafion binder by dissolution is a promising method to recover the GDL as is.

**Figure 4.**
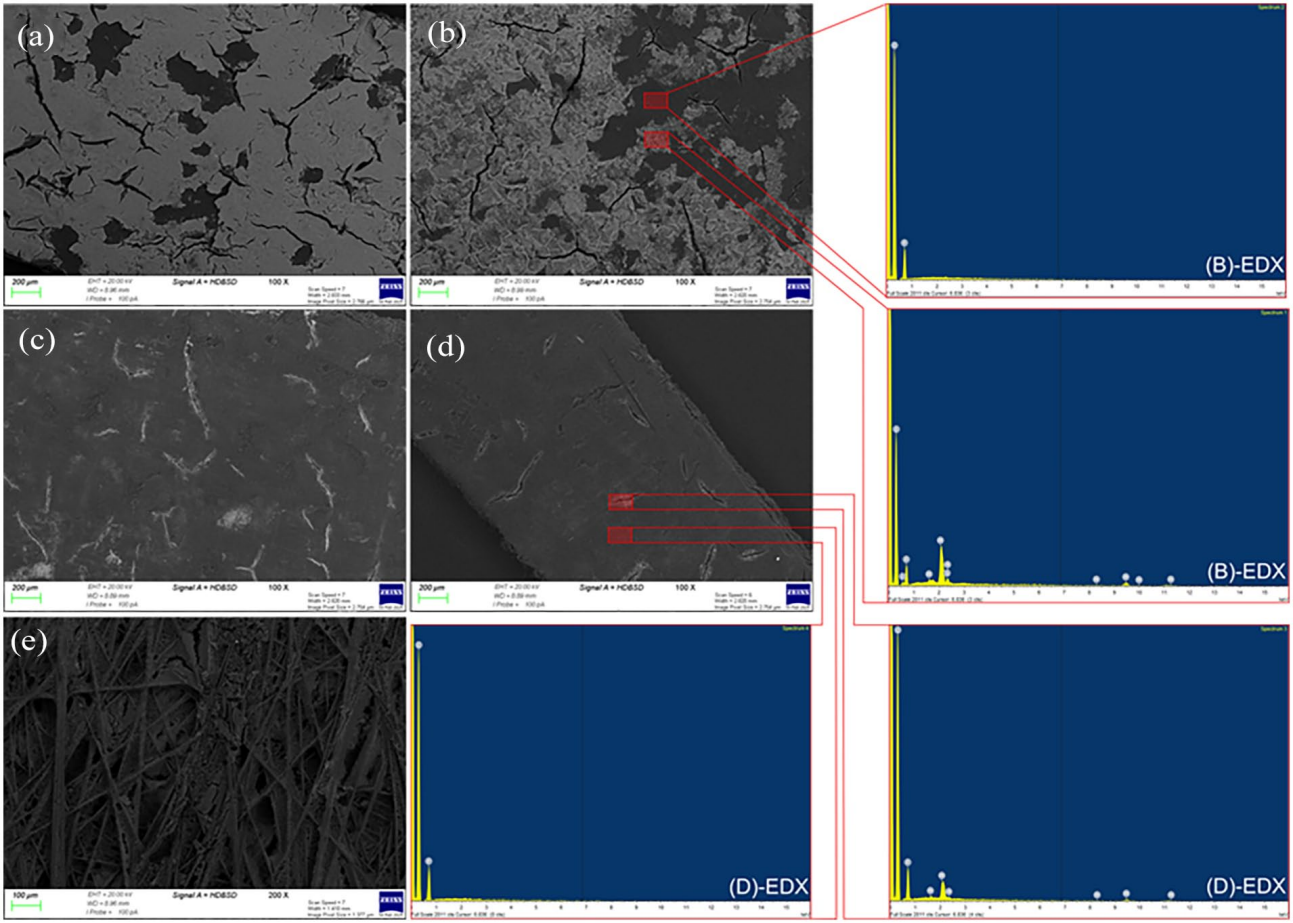
SEM-BSD images and associated EDX spectra of GDL microporous layer after microwave treatment at 90°C: (a) 5 minutes without stirring in 40% IPA solution, (b) 5 minutes with stirring in 40% IPA solution, (c) 15 minutes with stirring in 40% EtOH solution and (d) 15 minutes with stirring in 40% IPA solution. As well as the SEM-BSD image of macroporous layer after (e) 15 minutes with stirring in 40% IPA solution. EtOH: ethanol; IPA: isopropyl alcohol; GDL: gas diffusion layer; SEM-BSD: scanning electron microscopy with back scattered detector; EDX: energy dispersive X-ray.

A small increase in temperature to 90°C was explored by using a Flexiwave microwave reactor. The comparison was initially done with 40% IPA solution, a holding time of 5 minutes and without induced mixing. The FTIR-ATR measurement revealed an absorbance of 0.005 and is the same as that of boiling 40% IPA solution with 5 minutes holding time. Dissolution can be further enhanced by inducing mixing that led to a decrease in FTIR-ATR absorbance from 0.005 to 0.004 (see Supplemental Figure S6). The comparison of samples that have undergone microwave processing for with and without induced mixing was done by using SEM-BSD (Figure 4(a) and (b)). The EDX was used to help distinguish between the Pt/C–Nafion binder layer and the GDL microporous layer. Figure 4(a) reveals that the surface is largely comprised of Pt/C–Nafion binder (lighter colour), with localized areas revealing the underlying GDL microporous layer (darker colour) resulting from binder dissolution. Figure 4(b) reveals that the surface is still largely comprised of Pt/C–Nafion binder but with more homogeneous dissolution of the binder across the surface and revealing more GDL microporous layer underneath. The resultant mixing enhancement led to improved Nafion binder dissolution, which was subsequently used in all further studies. Additionally, the EDX of the different region reveals that the dissolution of the Nafion binder led to the liberation Pt/C catalyst.

From Figure 4, it can be seen that for a 40% IPA solution at 90°C, extending the holding time results in more Nafion binder being removed. Following 15 minutes holding time, the FTIR-ATR absorbance decreased to 0.002, and the GDL was left undamaged (Figure 4(d) and (e)). It was thought that an extended holding time would completely remove the Nafion binder. However, the FTIR-ATR absorbance from the 30-minute holding time also gave a value of 0.002 (Supplemental Figure S4). To understand this, a morphology analysis of GDL after microwave treatment was performed and is presented in Figure 4(d). The morphological analysis, supported by EDX spectral data, shows that Pt/C remains adhering near to the crack region, whereas other regions are relatively clean from Pt/C. It is important to point out that cracks are already present even from pristine GDL (see Supplemental Figure S7).

Although the EDX spectrum of the crack region did not register an elemental sulphur peak, the FTIR-ATR absorbance measurement was able to register detectable absorbance at the S–O stretching vibration region (Supplemental Figure S1C). Inferring to the results from the EDX and FTIR-ATR, it can be understood that the remaining Pt/C is still held together by the Nafion binder. Increasing the temperature shortens the holding time needed to dissolve the Nafion binder, but complete removal has been proven to be challenging.

The use of different EtOH and IPA concentrations between 10% and 80% were then conducted while maintaining the reactor temperature at 90°C and holding time constant at 15 minutes with the results summarized in Figure 3(a). It can be seen that, increasing the IPA and EtOH concentrations leads to more Nafion binder being removed. Moreover, it is important to point out that there was a minimum removal of Nafion binder with 10% EtOH solution and that the 20% IPA solution performs as well as the 40% EtOH solution. Contact angle measurements at various EtOH and IPA concentrations were then carried out to find the solvent composition that readily penetrates the GDL, with the results available in the Supplemental Table S1. The contact angle study reveals that 25% IPA solution (86.5° ± 0.8°) has a similar contact angle to that of 40% EtOH solution (87.0° ± 0.5°). This then helps explain why 20% IPA solution performs as well as 40% EtOH solution. Moreover, from the contact angle study, we found that 30% IPA solution instantaneously penetrates the GDL, whereas a 55% EtOH solution is required to have the same effect. Thus, the dissolution of Nafion binder experiences less mass transport limitation with IPA solution compared to the EtOH solution. This is reflected by the 40% IPA solution performing better than the 80% EtOH solution at dissolving the Nafion binder from the GDL.

From this study, the use of 40% IPA solution represents the optimum solvent for binder dissolution as it allows near complete dissolution of Nafion binder with 15 minutes holding time at 90°C. Further increasing the temperature to completely remove the Nafion binder was carried out, and the results are summarized in Figure 3(c). A temperature increase from 90°C to 150°C resulted in a decreased holding time of 5 minutes, compared to 15 minutes, in order to achieve an equivalent FTIR-ATR absorbance of 0.002 (Figure 3(c)). Further increasing the temperature did not lead to complete removal. Experiment employing a larger electrode (2.7 × 2.7 cm) carried out at 90°C and 15 minutes holding time successfully removed most of the Nafion binder, as evidenced by an FTIR-ATR absorbance of 0.002 (see Supplemental Figure S5). In addition, the elemental analysis reveals platinum distribution of 46% and 54% for the membrane cleaning and binder dissolution, respectively, and the potential platinum recovery rate was found to be 87% ± 6%. It is important to point out that the Nafion PEM is platinum-free, and the remaining 13% still resides within the GDL’s microporous layer.

### Direct recycling of the GDL

**Figure 5.**
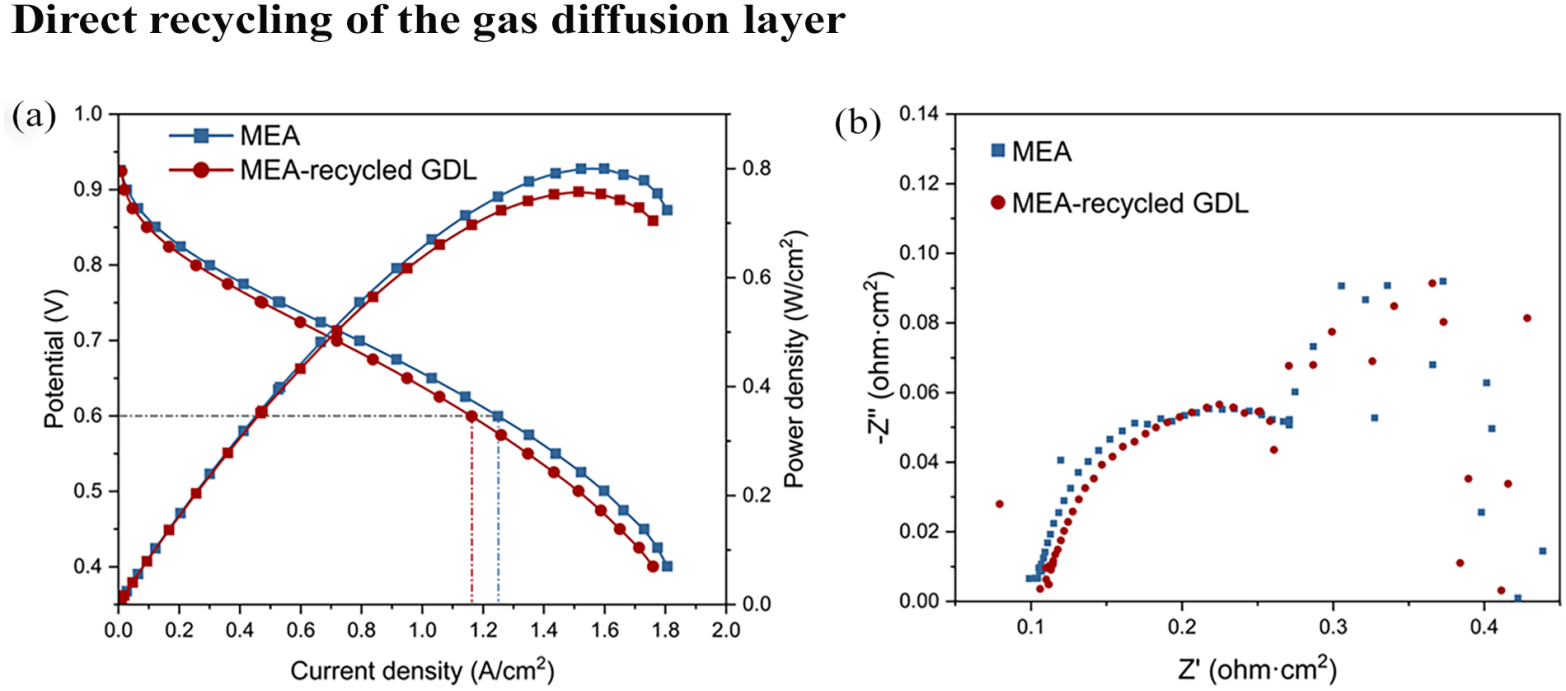
Comparison of the MEA fabricated from new GDL and recycled GDL through microwave treatment at 90°C and holding time of 15 minutes: (a) polarization curves and power density against current density and (b) impedance at 0.6 V. MEA: membrane electrode assembly; GDL: gas diffusion layer.

The recycled GDL was then assembled into an MEA and compared to MEA fabricated with pristine GDL, and the results are presented in Figure 5. Figure 5(a) shows a comparison of the polarization curves and power density variations. The MEA fabricated with the recycled GDLs performed well but less than that of the pristine GDL. The polarization curves show that at 0.6 V, the current density of the MEA with the recycled GDL decreased from 1.249 to 1.162 A cm^−2^ (c.a., 6.9%) compared to the pristine MEA. Similarly, there is a noticeable decrease in the peak power density from 0.800 to 0.758 W cm^−2^ (c.a., 5.3%). This decline in performance could be attributed to several factors inherent in the recycling process of the GDLs. The mechanical or thermal treatment of the process may alter the structural integrity or the electrochemical properties of the GDL material (Okonkwo and Otor, 2021). Changes in porosity, surface area or even the introduction of impurities could hinder electron and proton transport, thus impacting the overall efficiency of the electrochemical reaction (Wen et al., 2023). Additionally, any residual degradation products from the initial usage might adversely affect catalytic activity, leading to lower performance metrics in the MEA with the recycled GDL. Furthermore, the impedance of the GDLs was measured at 0.6 V with the results shown in Figure 5(b). It is evident that the MEA fabricated from the recycled GDL exhibits higher Ohmic resistance. This increase in resistance aligns with the findings from the polarization curves, where the recycled GDL MEA also showed decreased performance in terms of lower current density and power density.

## Conclusion

This study explores the possibility of direct recycling of PEM fuel cells. The sample uses Nafion as the PEM and Pt/C catalyst binder. Aqueous EtOH and IPA were used and compared to dissemble the MEA components by inducing Nafion membrane swelling. It was found that double the concentration of EtOH is required to that of 40% IPA solution to have the same separation effect within 24 seconds. Following the swelling procedure, Pt/C can be found on both Nafion membrane and GDL surface. Hence, Nafion membrane cleaning by sonication and GDL cleaning by dissolution were proposed. Similarly, to the swelling, various aqueous EtOH and IPA solutions were compared for sonication efficacy. From the sonication study, it was found that the Pt/C on the Nafion membrane can be liberated after 5 minutes sonication with 40% IPA. Moreover, the sonication procedure has been estimated to incur a Nafion membrane mass loss of 2.0% ± 1.2%.

The use of aqueous alcohol at elevated temperatures was explored to dissolve the Nafion binder on GDL. A significant reduction in the relative abundance of Nafion binder on the GDL was achieved by boiling the electrode in 40% IPA for 20 minutes, as shown by the FTIR-ATR absorbance at 983 cm^−1^ that significantly decreased from 0.038 to 0.003. To understand the effect of elevated temperature on the dissolution process, a microwave reactor was used. A slight increase in temperature from boiling the 40% IPA (ca., 83.3°C) to 90°C with a holding time of 15 minutes allows better dissolution than boiling 40% IPA for the same holding time. Various concentrations of EtOH and IPA, between 10% and 80%, at 90°C and 15 minutes holding time were compared. The results suggest that almost no removal of Nafion occurs with 10 % EtOH, whereas 20% IPA works as well as 40% EtOH. A contact angle study of various EtOH and IPA was done to understand this phenomenon, and it was found that aqueous IPA is better at wetting the GDL compared to the aqueous EtOH. The 40% IPA solution gave the best balance between concentration and Nafion removal as further increases in IPA concentration did not lead to more Nafion dissolution. It was found that the holding time can be shortened to 5 minutes at temperature above 150°C. However, the complete removal of Nafion on the GDL remains challenging regardless of the temperature.

The platinum recovery was found to be 87% ± 6% with the incomplete liberation of platinum caused by the incomplete dissolution of Nafion. SEM-BSD revealed that the catalyst is mainly found concentrated near to the crack on the GDL, with the finding further supported with EDX spectrum taken from the area. The recovered GDL was fabricated into a new MEA and tested for its performance. It was found that there is a slight reduction in performance shown by the 6.9% lower current density and 5.3% peak power density. This study demonstrates the possibility of recovering clean Nafion membrane with minimum mass loss and direct recycling of GDL. The application of microwave heating is deemed suitable, given that a slight increase in temperature and pressure suffices to achieve GDL cleaning. The direct recycled GDL showed a slight degradation in performance due to incomplete dissolution of Nafion binder. However, the cost associated with the proposed recycling technique still needs to be assessed by considering the solvent reusability, the cost of processing recovered platinum to a catalyst grade and the recovered Nafion membrane reusability (e.g. assessment of gas crossover). Future work should be focused on recovering the dissolved Nafion binder as well as understanding the effect of the recycling cycles and parameters towards GDL performance and ways to attain 100% Pt/C recovery by complete dissolution of the Nafion.

The development of PEM fuel cells is an active area of research, with a focus on improving MEA durability by optimizing catalyst structure, support selection and processing techniques. Optimal design balances durability with sustainability. Consequently, future MEA designs should integrate end-of-life materials recovery and recycling strategies. This outcome is achievable through collaborative materials and recycling research.

## Supplemental Material

sj-docx-1-wmr-10.1177_0734242X251360546 – Supplemental material for Have proton exchange membrane fuel cells been designed for recycling? Supplemental material, sj-docx-1-wmr-10.1177_0734242X251360546 for Have proton exchange membrane fuel cells been designed for recycling? by Samuel D Widijatmoko, Yichang Yan, Qiqiu Huang, Shangfeng Du, Yongliang Li and Gary A Leeke in Waste Management & Research
