# Supplementary material for: Have proton exchange membrane fuel cells been designed for recycling?
Source: Waste Manag Res. 2025 Aug 18;43(10):1467–75. doi: 10.1177/0734242X251360546 (PMC12476485; doi:10.1177/0734242X251360546)
Supplement: sj-docx-1-wmr-10.1177_0734242X251360546 – Supplemental material for Have proton exchange membrane fuel cells been designed for recycling? [file sj-docx-1-wmr-10.1177_0734242X251360546.docx]

Supplementary information for

**Have proton exchange membrane fuel cells been designed for recycling?**

Samuel D. Widijatmoko, Yichang Yan, Qiqiu Huang, Shangfeng Du, Yongliang Li, Gary A. Leeke

School of Chemical Engineering, University of Birmingham, Birmingham B15 2TT, United Kingdom


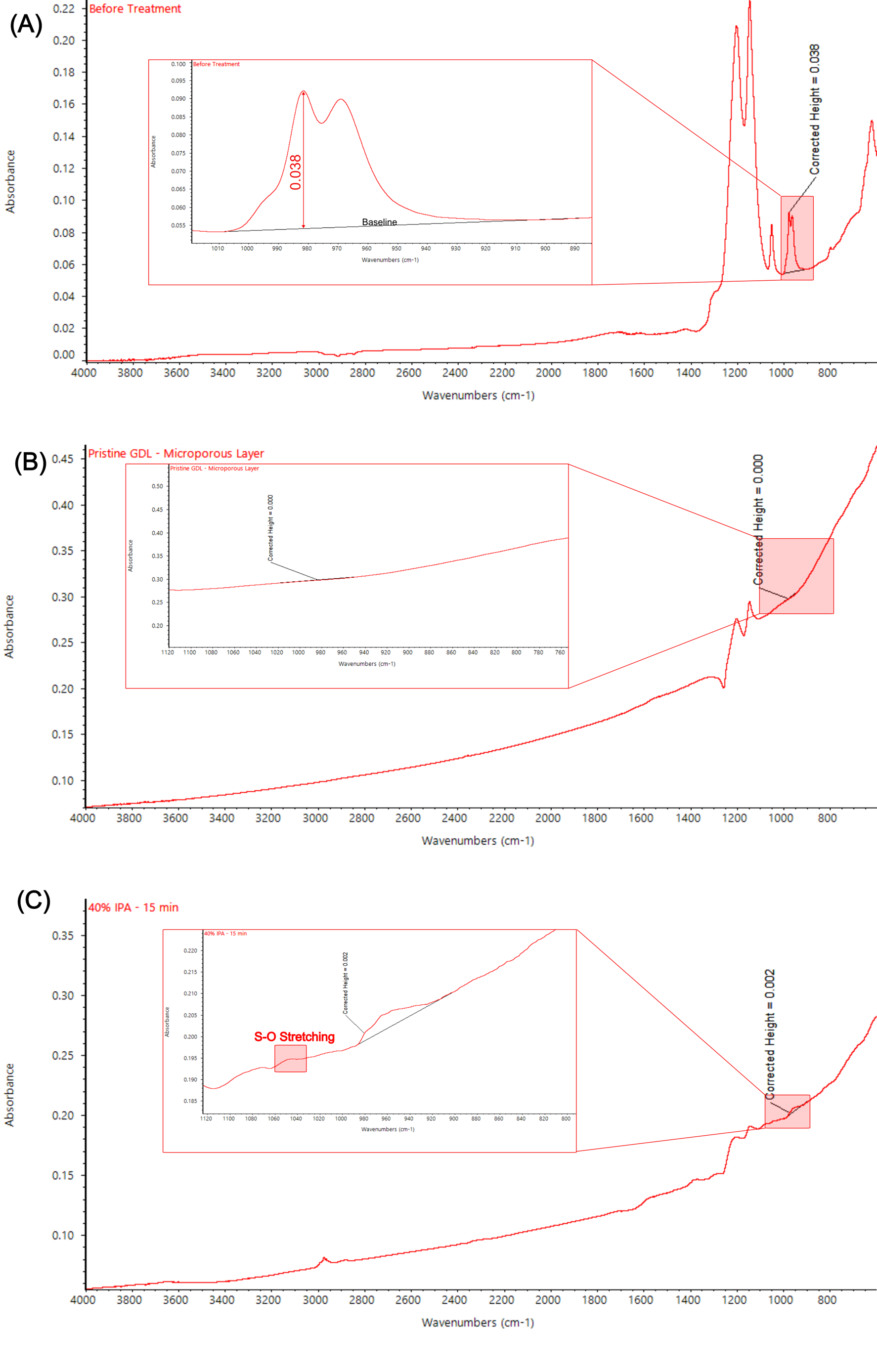


Figure S 1 – FTIR-STR spectrum taken from GDL’s microporous side with corrected absorbance of (A)GDL before treatment, (B) New GDL and (C) GDL after microwave treatment at 90^o^C with 15 min holding time.


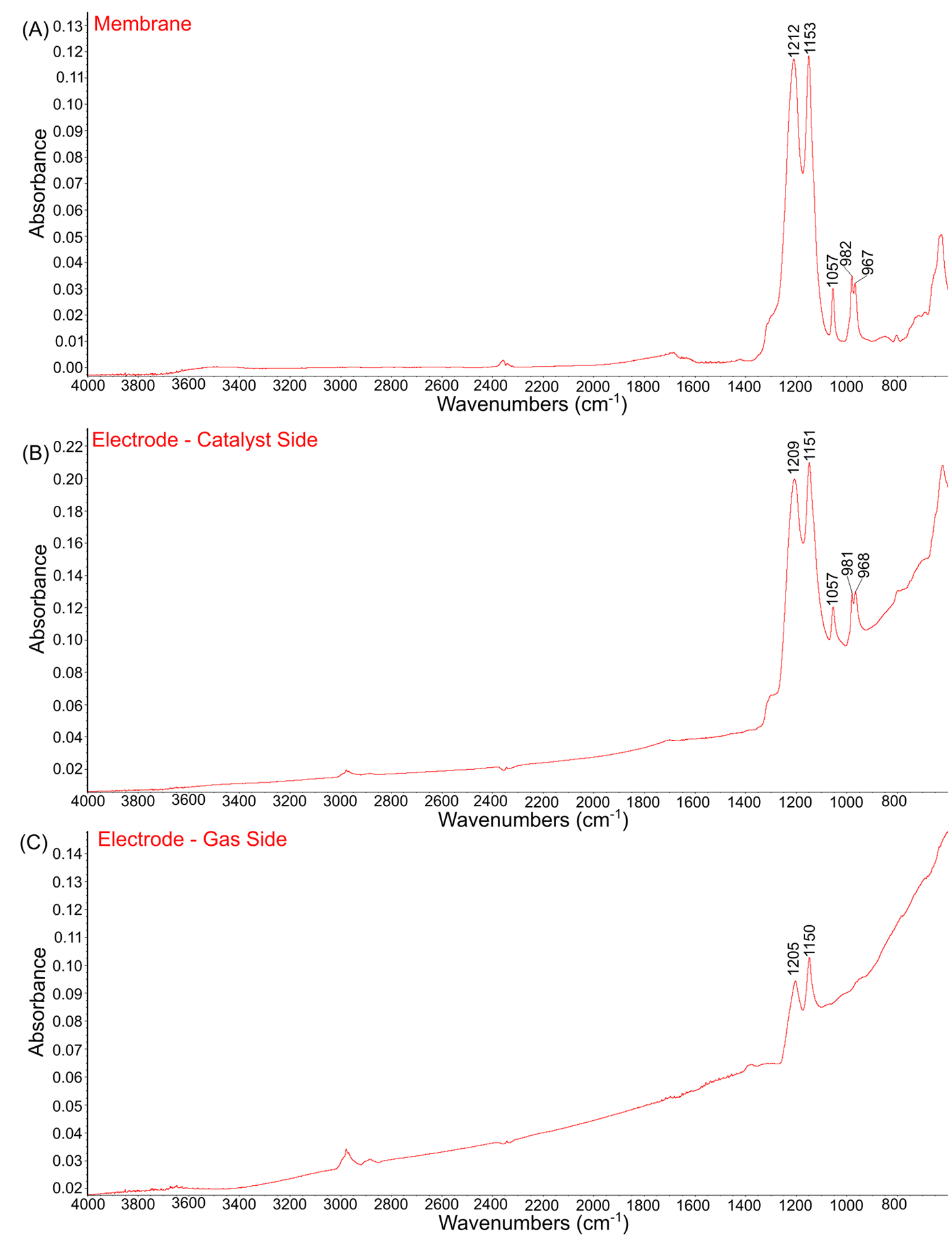


Figure S 2 – FTIR-ATR spectrum of (A) PEM, (B) Electrode – Catalyst Side and (C) Electrode – Gas Side.

Table S 1 - Contact angle measured on the GDL’s macroporous side for EtOH and IPA at various concentrations.

| Concentration  (%) | Contact Angle  (θ) |
| --- | --- |
| Ethanol | |
| 0 | 142.9 ± 0.9 |
| 10 | 131.6 ± 0.5 |
| 20 | 112.6 ± 0.7 |
| 40 | 87.0 ± 0.5 |
| 50 | 79.0 ± 0.6 |
| 55 | Absorbed |
| Isopropanol | |
| 10 | 129.9 ± 0.4 |
| 20 | 107.2 ± 0.6 |
| 25 | 86.5 ± 0.8 |
| 30 | Absorbed |


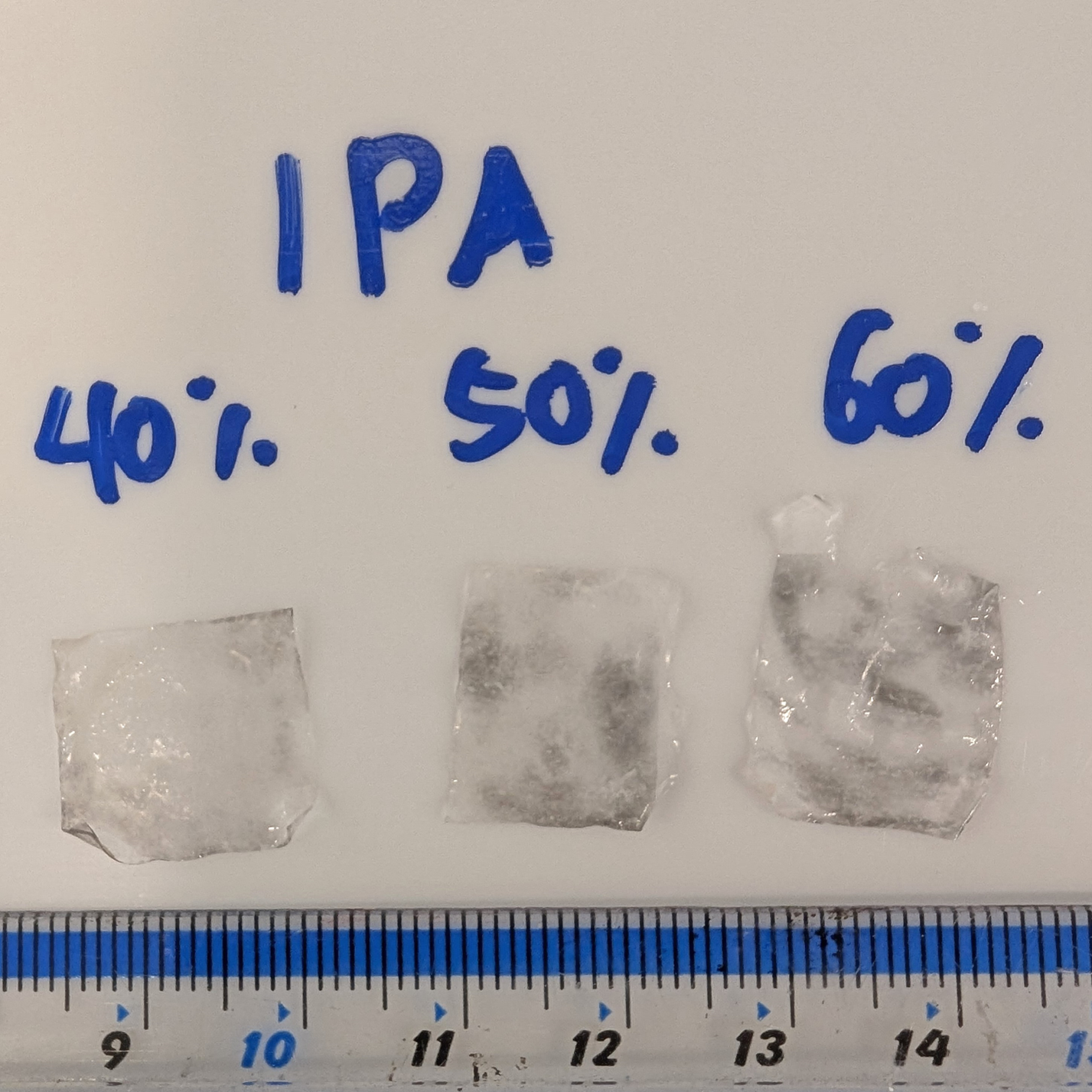


Figure S 3 – Comparison of membrane after 5 min sonication with various concentration of IPA solution.


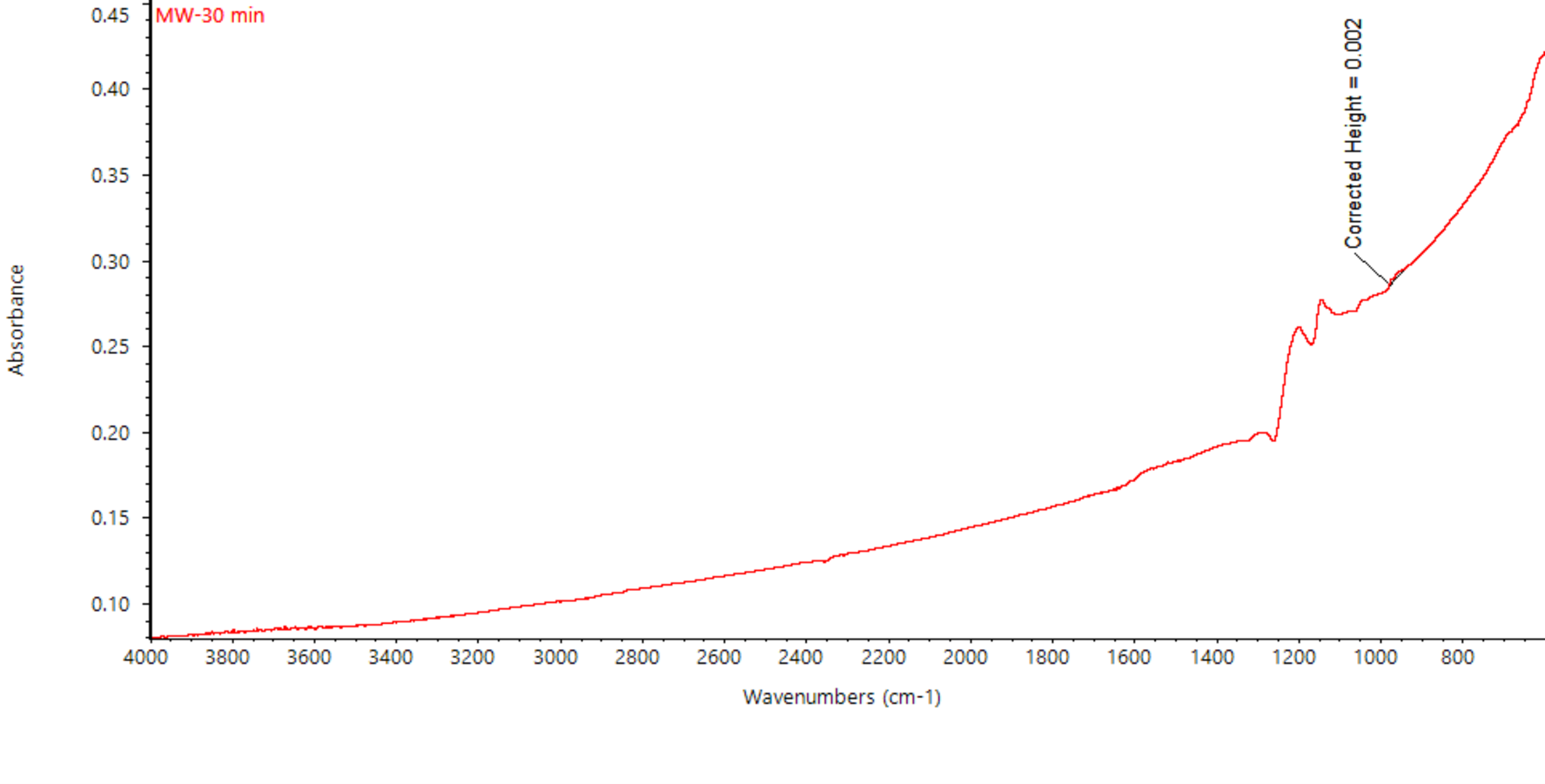


Figure S 4 – FTIR-ATR spectrum of a sample after 30 min holding time at 90^o^C.


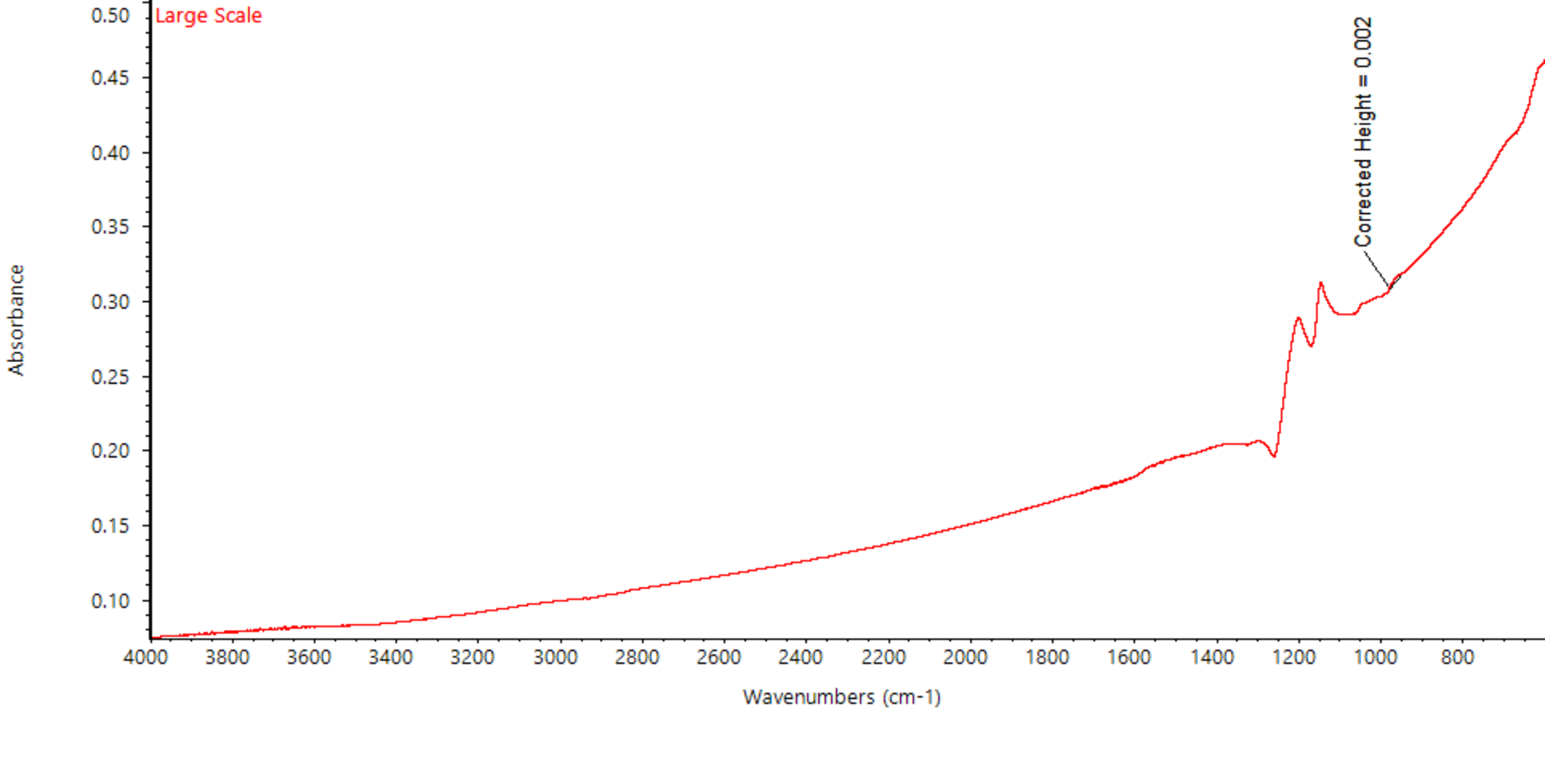


Figure S 5 – FTIR-ATR spectrum of large-scale sample after 15 min holding time at 90^o^C.


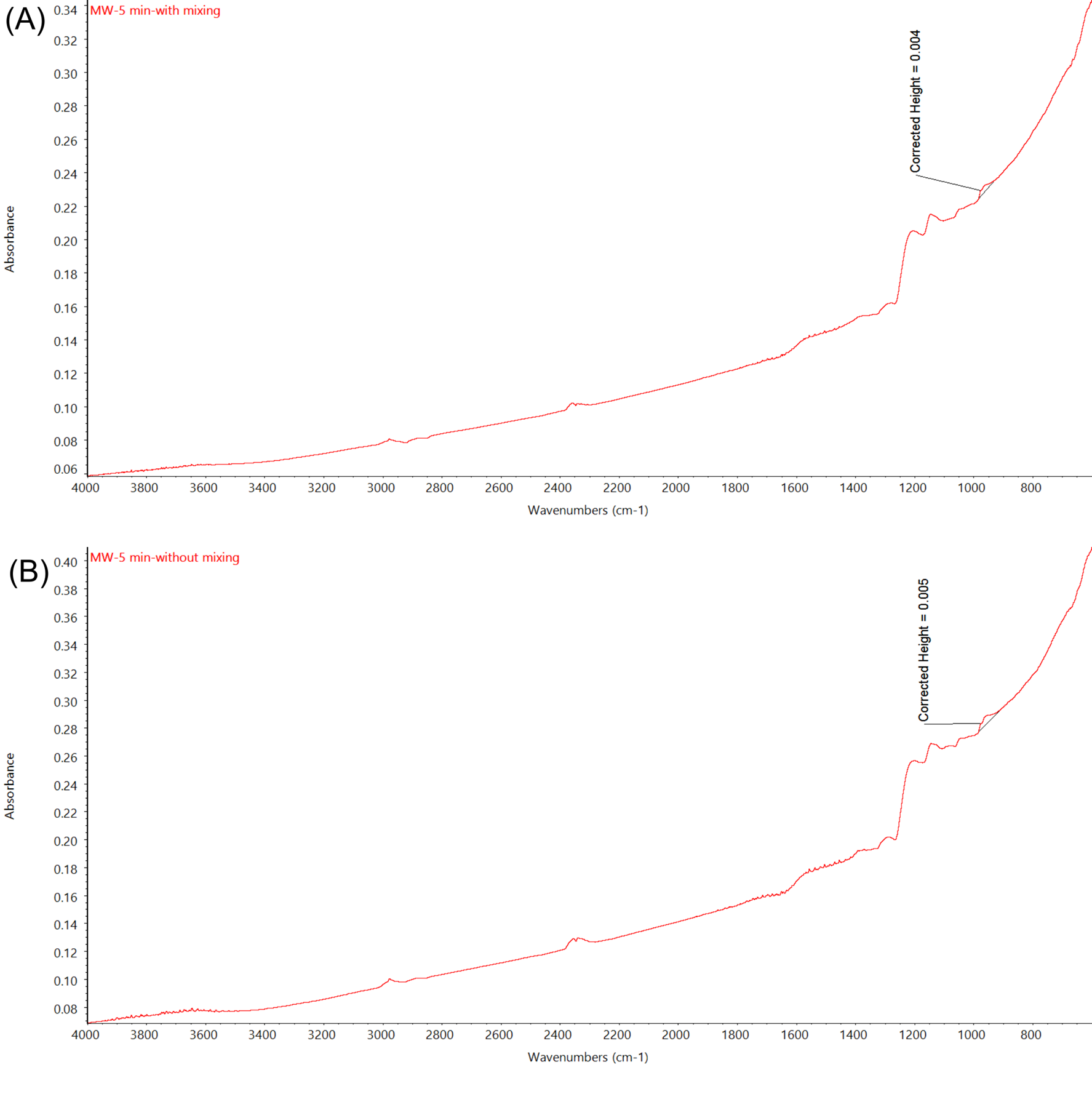


Figure S 6 – Comparison of FTIR-ATR spectrum of samples that have undergone microwave treatment at 90^o^C with 5 min holding time of treatment (A) with mixing and (B) without mixing.


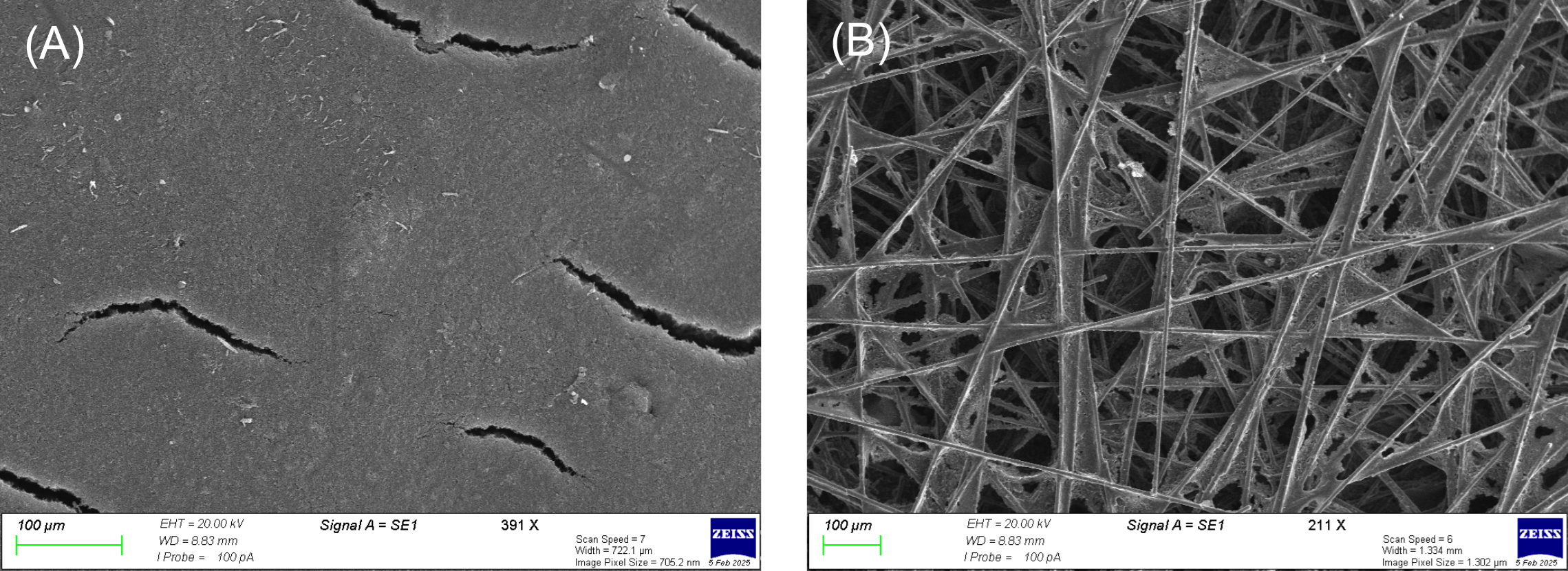


Figure S 7 - SEM image of pristine GDL (A) microporous layer and (B) macroporous layer.
